# Blood Plasma Analysis in Ovarian Cancer Patients Using an AFM/MS Approach: Effect of Sample Dilution on Proteome Depth

**DOI:** 10.3390/ijms27177541

**Published:** 2026-08-23

**Authors:** Arina I. Gordeeva, Anastasia A. Konstantinova, Tatiana A. Materova, Elizaveta E. Rybakova, Maria O. Ershova, Nikita E. Vavilov, Victor G. Zgoda, Tatyana O. Pleshakova, Alexander I. Archakov

**Affiliations:** Institute of Biomedical Chemistry (IBMC), 119121 Moscow, Russia; varuevavarueva@gmail.com (A.A.K.); materova.ta20@physics.msu.ru (T.A.M.); zx2405@yandex.ru (E.E.R.); motya00121997@mail.ru (M.O.E.); n.vavilov95@gmail.com (N.E.V.); victor.zgoda@gmail.com (V.G.Z.); alexander.archakov@ibmc.msk.ru (A.I.A.)

**Keywords:** atomic force microscopy, plasma proteomics, protein immobilization, photoactive crosslinker, dilution effect, mass spectrometry, dynamic range

## Abstract

Early detection of ovarian cancer remains challenging because of the lack of sensitive and reproducible blood-based biomarkers. A major challenge in plasma proteomics is the extremely wide dynamic range of protein concentrations, which prevents simultaneous detection of both high- and low abundance proteins and limits the identification of disease-associated signals. In this study, we applied a combined atomic force microscopy and mass spectrometry (AFM/MS) approach to investigate how sample dilution affects plasma proteome coverage and the detection of differences between healthy donors and patients with stage I and stage III ovarian cancer. Plasma samples were analyzed at two dilution levels (1:100 and 1:10,000). At 1:100 dilution, a total of 235 proteins were identified across all samples, representing the union of all replicates and groups. The reproducible CORE proteome comprised 169 proteins in the Healthy group, 183 in the Stage I group, and 193 in the Stage III group. Differential analysis revealed distinct, non-overlapping protein sets at each dilution level. At 1:100 dilution, most altered proteins were decreased in patients and corresponded to major plasma components, including complement proteins and protease inhibitors. At 1:10,000 dilution, most altered proteins were increased and were predominantly immunoglobulin-related proteins, along with complement regulatory components. These findings show that sample dilution determines which fraction of the plasma proteome is observable. Here, proteome depth refers to the total number of non-redundant proteins accessible within the analytical workflow. When CORE sets from all groups were combined, 216 proteins were identified at 1:100 and 149 at 1:10,000, with 133 shared between the two dilution conditions. The higher dilution contributed 16 additional CORE proteins not detected in the 1:100 CORE union, increasing the combined CORE set to 232 proteins. Thus, higher dilution alone did not increase proteome depth, but provided complementary protein identifications that increased cumulative proteome depth when both dilution conditions were considered together. This effect reflects dilution-dependent selectivity in the composition of the detectable protein subset.

## 1. Introduction

Ovarian cancer remains one of the most lethal gynaecological malignancies worldwide [1]. Five-year survival rates for ovarian cancer vary greatly by stage [2]. At stage I, they exceed 90%, while at stage IV, survival drops to 20%. This large difference can be explained by the greater aggressiveness of advanced-stage tumors and by the fact that diagnosis is often delayed [3]. Women typically seek medical attention only after symptoms appear, and as a result the disease is detected at stages I and II in fewer than one-third of patients. There is still no approved ovarian cancer screening method that is accurate enough for widespread use [4]. CA125 remains the main blood-based biomarker used in the clinical management of ovarian cancer. It is used for diagnosis in postmenopausal women and for treatment monitoring. However, the sensitivity and specificity of CA125 are insufficient [5]. Due to low specificity, many women with benign lesions undergo unnecessary surgeries. For example, in Sweden, 69.7% (445/638) of women operated on for suspected ovarian cancer ultimately had a benign tumor, while the remaining cases included borderline (*n* = 31) and malignant (*n* = 162) tumours [6].

To improve accuracy, combinations of markers have been developed. The Risk of Ovarian Malignancy Algorithm (ROMA) combines CA125 and HE4 biomarkers with menopausal status to generate a composite risk score. The ROMA is a valuable diagnostic tool, particularly for postmenopausal women, providing higher accuracy than CA125 alone. In this group, sensitivity reaches 93.75% and specificity 94.9%. However, in premenopausal women, ROMA’s sensitivity is limited [7]. The FDA-approved OVA1 (ApoA1, β2-microglobulin, CA125, transferrin, transthyretin) test demonstrated a sensitivity of 96% and a specificity of 28% in postmenopausal women and a sensitivity of 85% and specificity of 40% in premenopausal women, and detected 76% of malignancies missed by CA125 [8]. However, the specificity rates for OVA1 are too low for accurate screening.

It should be emphasized that ROMA and OVA1 are intended for the preoperative assessment of an adnexal mass in women already scheduled for surgery, and are not labeled by the FDA for population screening. Because ovarian cancer is rare, early-stage screening should provide the highest possible specificity and sensitivity: 99.6% and 75%, respectively. This is necessary to minimize false-positive results [9].

Mass spectrometry (MS)-based proteomics is considered a key approach to cancer biomarker discovery [10,11]. This method enables large-scale analysis of protein abundance, post-translational modifications, and signaling pathways [12]. In ovarian cancer, proteomic methods have been applied to tumor tissue, ascitic fluid, serum, and plasma. This has helped identify changes associated with progression, prognosis, and response to therapy [13].

Blood plasma is particularly convenient as a source of biomarkers because it can be obtained by minimally invasive procedures and reflects systemic pathological processes [14]. However, analysis of plasma proteomes has serious limitations. This is due to the very wide dynamic range of protein concentrations: from albumin (approximately 0.5–0.8 mM) to signaling proteins present at concentrations below 10^−15^ M. This difference is more than ten orders of magnitude [15,16]. According to Metatla et al. [17], just 22 of the most abundant proteins account for 99% of the total mass of plasma proteins. Albumin alone accounts for approximately half of this mass.

Experimental studies using the LTQ Orbitrap hybrid mass spectrometer have shown that, within a single spectrum (intraspectrum dynamic range), an intensity range of approximately 10^4^ can be achieved; that is, the strongest and weakest detectable ions can differ by a factor of 10,000 [18]. Direct non-targeted analysis (shotgun LC-MS/MS) of plasma without pre-enrichment faces a major limitation: its dynamic range (10^4^–10^5^) is insufficient to simultaneously detect both high- and low abundance proteins [19]. Therefore, in a standard proteomic experiment, only hundreds of the most abundant components are identified, while signals from low-concentration proteins remain below the detection limit. Several enrichment strategies have been developed to address this limitation. Immunodepletion (MARS, Seppro IgY14, and Pierce columns) removes the 6–20 most abundant proteins with >90% efficiency, but is expensive and can non-specifically deplete low-abundance proteins. Combinatorial peptide libraries (ProteoMiner) compress the dynamic range through saturable binding: highly abundant proteins are removed, while low-abundance ones are enriched [20]. Chemical precipitation (ammonium sulfate and polyethylene glycol) is inexpensive but non-specific. Pre-fractionation (electrophoresis and chromatography) reduces sample complexity at the expense of time and material consumption. All these methods can result in the loss of target proteins and reduced reproducibility. Recent developments (nanofibers, imprinted polymers, cryogels) have not yet been commercialized [21].

A complement to the above methods is the use of flat functionalized surfaces for the immobilization and concentration of plasma proteins. Unlike bulk chromatographic supports, flat substrates (e.g., silicon, mica, or gold) not only allow for the efficient adsorption of proteins from the analyzed sample but also their direct visualization using atomic force microscopy (AFM) prior to mass spectrometric identification. This approach enables the assessment of the distribution density, morphology, and size of immobilized protein particles at the nanometer level, which is unattainable with traditional enrichment in columns or on beads. Furthermore, flat surfaces can be chemically modified to selectively capture specific classes of proteins (e.g., through affinity ligands or hydrophobic interactions) [22].

In this study, we further develop an approach based on the use of chips modified by 4-benzoylbenzoic acid succinimidyl ester (SuccBB) for the concentration of plasma proteins. We previously demonstrated the effectiveness of such photoactivatable surfaces for protein immobilization followed by mass spectrometric identification [23,24,25]. In the present study, we applied this technology to the comparative analysis of clinical samples from patients diagnosed with ovarian cancer. Our goal is to study the protein composition of plasma in patients at early (stage I) and late (stage III) stages of the disease. Data from patients with ovarian cancer were compared with previously published data from healthy volunteers [25]. A small cohort was selected to enable an initial assessment of the developed AFM/MS approach and its potential to detect differences in proteomic profiles between healthy donors and patients. Although the data obtained are preliminary, they establish a foundation for future research involving larger sample sizes, which will require the validation of identified proteins using independent methods (such as ELISA or MRM). We have previously demonstrated that incorporating an on-chip concentration step—using AFM chips—enables the identification of a greater number of proteins compared to direct plasma analysis. The advantage of flat mica chips lies in the ability to perform all analytical steps sequentially on a single substrate with an area of approximately 1 cm^2^—including surface modification, sample incubation, AFM imaging, and enzymatic hydrolysis—thereby minimizing sample loss and the risk of contamination. The atomically smooth surface allows for the monitoring of all modification stages and protein layer formation via AFM, while the localization of proteins within a confined area ensures a high concentration of proteins at the surface. We plan to expand the scope of our AFM data in future work and anticipate that the developed approach will serve as an effective tool for comparative proteomic studies.

## 2. Results

### 2.1. AFM Imaging Results

**Figure 1:**
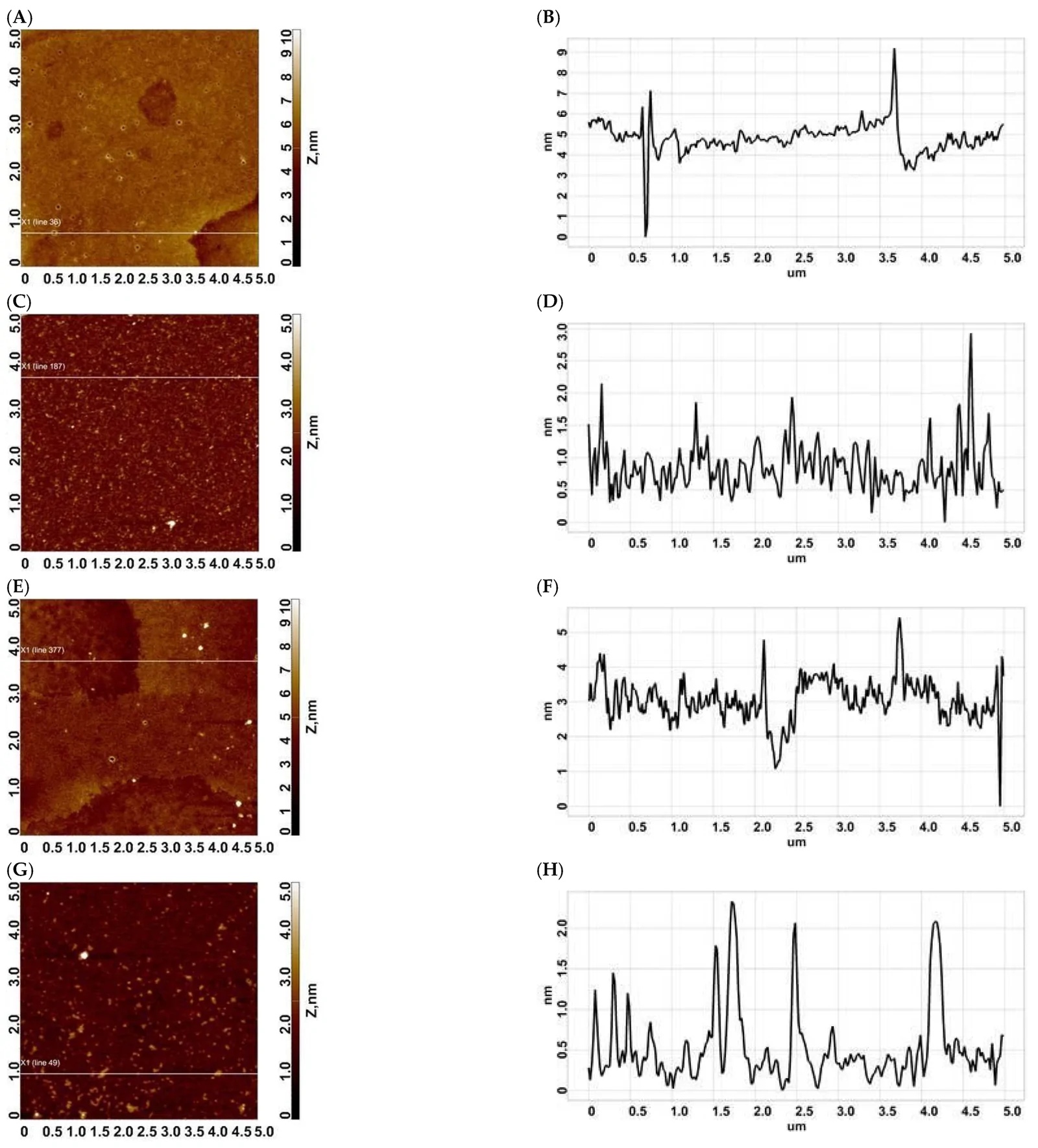
Typical AFM images of the surface of modified mica after incubation in plasma of patients with stages I and III: (**A**,**B**)—stage I, dilution 1:100 (scan and section); (**C**,**D**)—stage I, dilution 1:10,000 (scan and section); (**E**,**F**)—stage III, dilution 1:100 (scan and section); (**G**,**H**)—stage III, dilution 1:10,000 (scan and section). The size of AFM images is 5 × 5 µm^2^.

Atomic force microscopy (AFM) was used to characterize the morphology of the adsorbed protein layer formed on chip surfaces after incubation in diluted plasma. The surface contained layer fragments, pores, compact rounded objects, and multilayer regions. Figure 1 shows AFM images and corresponding height profiles for plasma samples from patients with stage I and III ovarian cancer at dilutions of 1:100 and 1:10,000.

As shown in Figure 1, for a dilution of 1:100 in both cases (stage I (A, B) and stage III (E, F)), pores with depths of 4–6 nm, fragments of a layer with a height of ~2 nm, as well as compact objects with different heights are visualized on the surface.

In the case of a 1:10,000 dilution, fragments with heights of 1–3 nm are predominantly visualized on the surface after incubation in plasma of patients with stages I and III of ovarian cancer. Additional AFM surface images are provided in Figure S1 (Supplementary Materials).

### 2.2. Analysis of the CORE Proteome and Molecular Mass Distribution

#### 2.2.1. CORE Proteome Across Dilution Regimes

To delineate the reproducible part of the plasma proteome in AFM/MS-MS measurements, the analysis was restricted to CORE proteins, defined as proteins consistently identified across all technical replicates within each group.

At 1:100 dilution, CORE proteome sizes were 169, 183 and 193 proteins in the Healthy, Stage I and Stage III groups, respectively. Increasing the dilution to 1:10,000 was associated with a reduction in CORE size to 120, 99 and 111 proteins.

**Figure 2:**
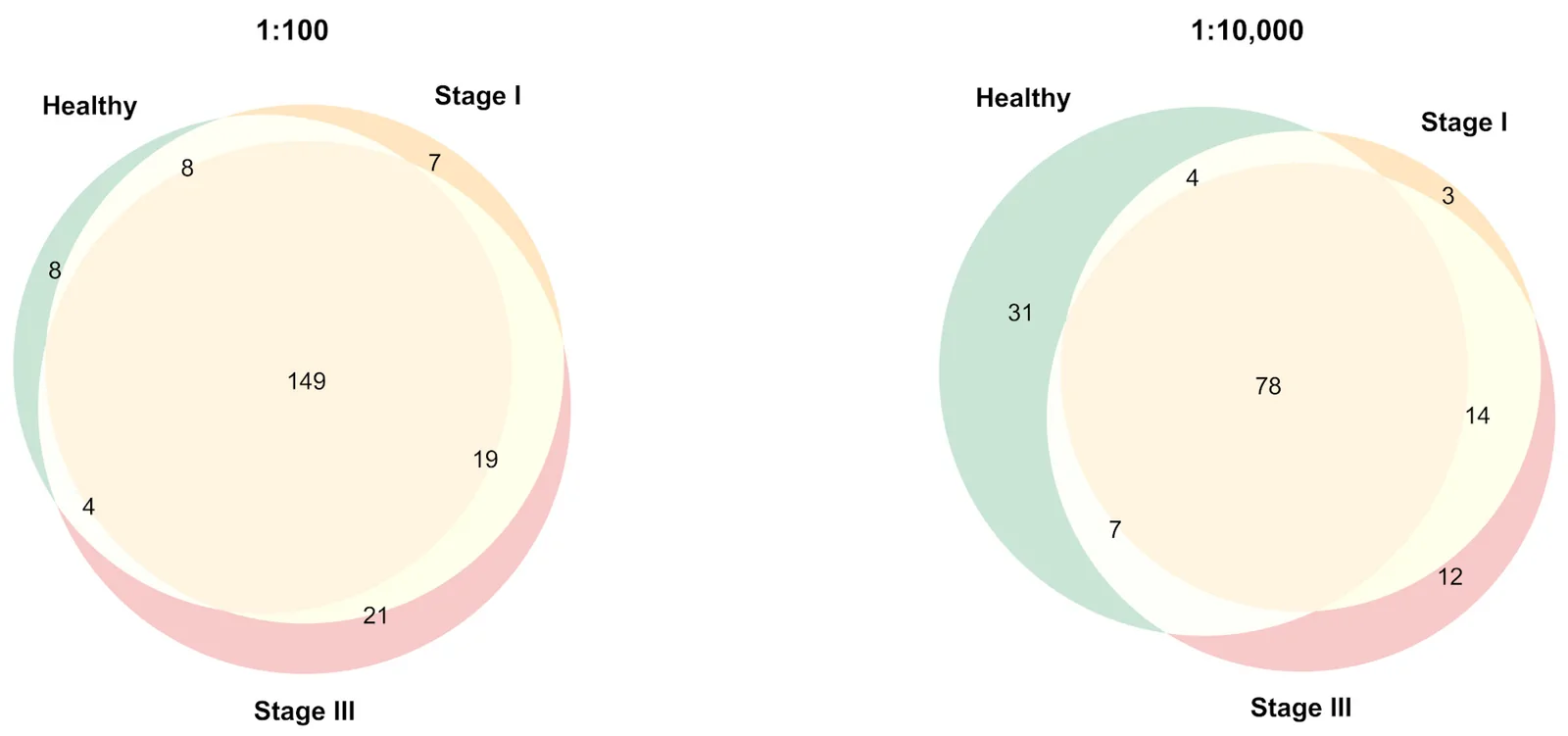
Overlap of CORE proteomes across dilution regimes. Venn diagrams illustrate the overlap of CORE proteins (proteins detected in all technical replicates) among Healthy, Stage I and Stage III groups at 1:100 and 1:10,000 dilution. Color coding: Green—Healthy, Beige—Stage I, Pink—Stage III.

A substantial subset of proteins remained common to all groups, comprising 149 proteins at 1:100 and 78 proteins at 1:10,000, indicating the presence of a stable reproducible component of the plasma proteome (see Figure 2).

At 1:100 dilution, the number of group-specific CORE proteins was limited (Healthy: 8; Stage I: 7; Stage III: 21). At 1:10,000 dilution, the number of group-specific proteins increased (Healthy: 31; Stage I: 3; Stage III: 12), concomitant with a decrease in the shared CORE.

Collectively, these results indicate that increasing dilution is associated with a reduction in the number of proteins consistently detected across technical replicates and a relative increase in group-specific CORE components.

When CORE sets from the Healthy, Stage I, and Stage III groups were pooled within each dilution condition, the CORE union comprised 216 proteins at 1:100 and 149 proteins at 1:10,000. Of these, 133 proteins were shared between dilutions, whereas 16 CORE proteins were detected only at 1:10,000. Combining both dilution conditions therefore increased the cumulative CORE union from 216 to 232 proteins, corresponding to a 7.4% increase relative to the 1:100 condition alone.

Stage I ovarian cancer is of particular relevance to early detection. However, the small Stage I cohort (*n* = 5) limits the statistical power for a dedicated differential abundance analysis. Stage I-specific changes were therefore not analyzed further in the present study and should be investigated in a larger independent cohort. The differential abundance analysis presented below was restricted to the Stage III versus Healthy comparison.

At 1:100 dilution, a substantial shared CORE (149 proteins) is observed across all groups, with a limited number of group-specific proteins (Healthy: 8; Stage I: 7; Stage III: 21).

At 1:10,000 dilution, the shared CORE decreases to 78 proteins, while the number of group-specific CORE proteins increases (Healthy: 31; Stage I: 3; Stage III: 12).

#### 2.2.2. Molecular Mass Distribution of CORE Proteins

**Figure 3:**
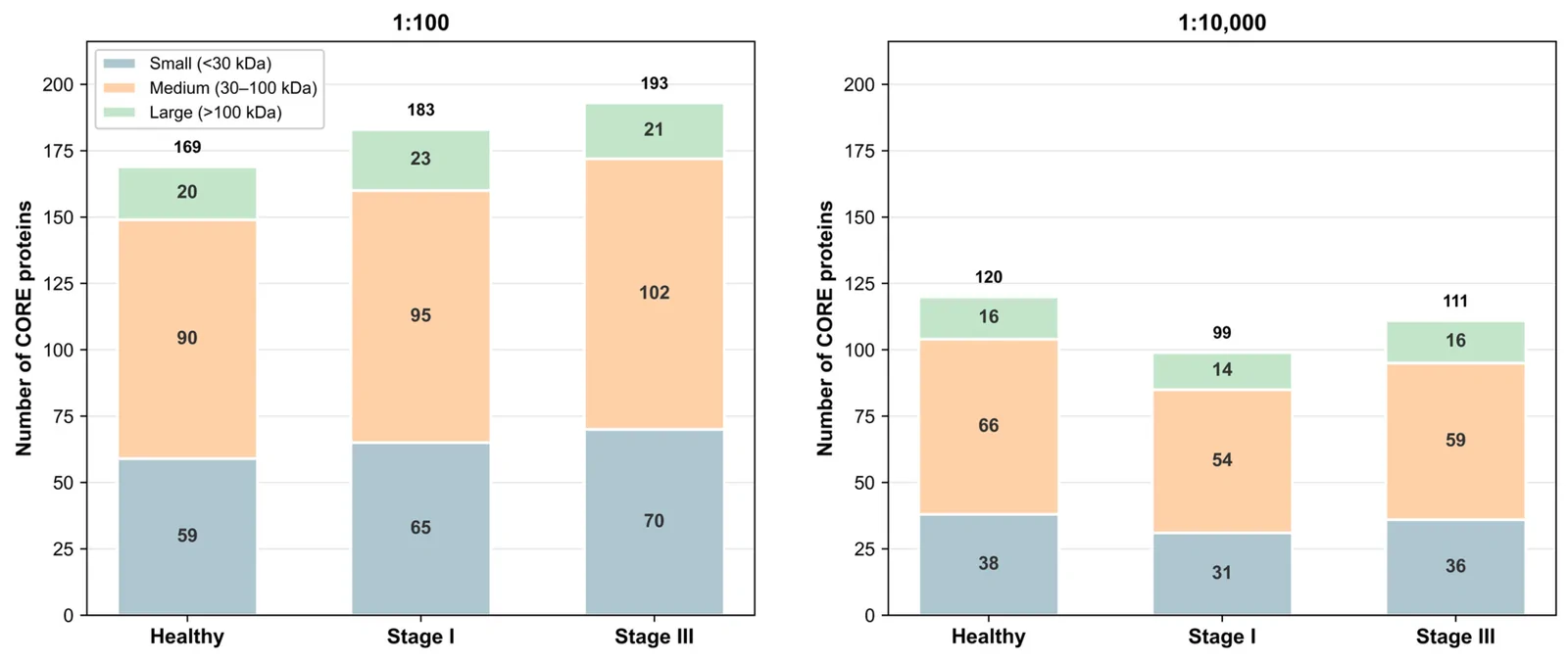
Molecular mass distribution of CORE proteins in MS analysis. Stacked bar plots showing the distribution of CORE proteins across molecular mass categories (<30 kDa (blue), 30–100 kDa (orange), >100 kDa (green)) for each group and dilution condition.

The molecular mass distribution of CORE proteins was evaluated for each group and dilution condition (Figure 3).

At 1:100 dilution, the distribution of molecular masses was comparable across groups. Proteins in the 30–100 kDa range constituted the majority of the CORE (~52–53%), whereas proteins <30 kDa and >100 kDa accounted for ~35–36% and ~11–13%, respectively.

At 1:10,000 dilution, the total number of CORE proteins decreased across all groups. The relative contribution of proteins <30 kDa was reduced, whereas the proportion of proteins in the 30–100 kDa and >100 kDa ranges showed a modest increase. In parallel, the mean molecular mass increased in all groups (Healthy: 60.4 to 62.8 kDa; Stage I: 62.3 to 64.2 kDa; Stage III: 57.8 to 64.6 kDa).

The absolute number of proteins in the 30–100 kDa range decreased upon dilution:

1. Healthy: 90 to 66;
2. Stage I: 95 to 54;
3. Stage III: 102 to 59.

Taken together, these observations indicate a dilution-dependent shift in the composition of the subset of proteins consistently detected across replicates, accompanied by a redistribution of molecular mass categories.

At 1:100 dilution, the distribution is comparable across groups, with dominance of proteins in the 30–100 kDa range. At 1:10,000 dilution, the total number of CORE proteins decreases, the proportion of low-molecular-weight proteins is reduced, and the mean molecular mass increases across all groups. Mass and reference abundance annotations for all quantified proteins are provided in Table S2 (Supplementary Materials).

#### 2.2.3. Distribution of Protein Concentrations in the CORE Proteome

**Figure 4:**
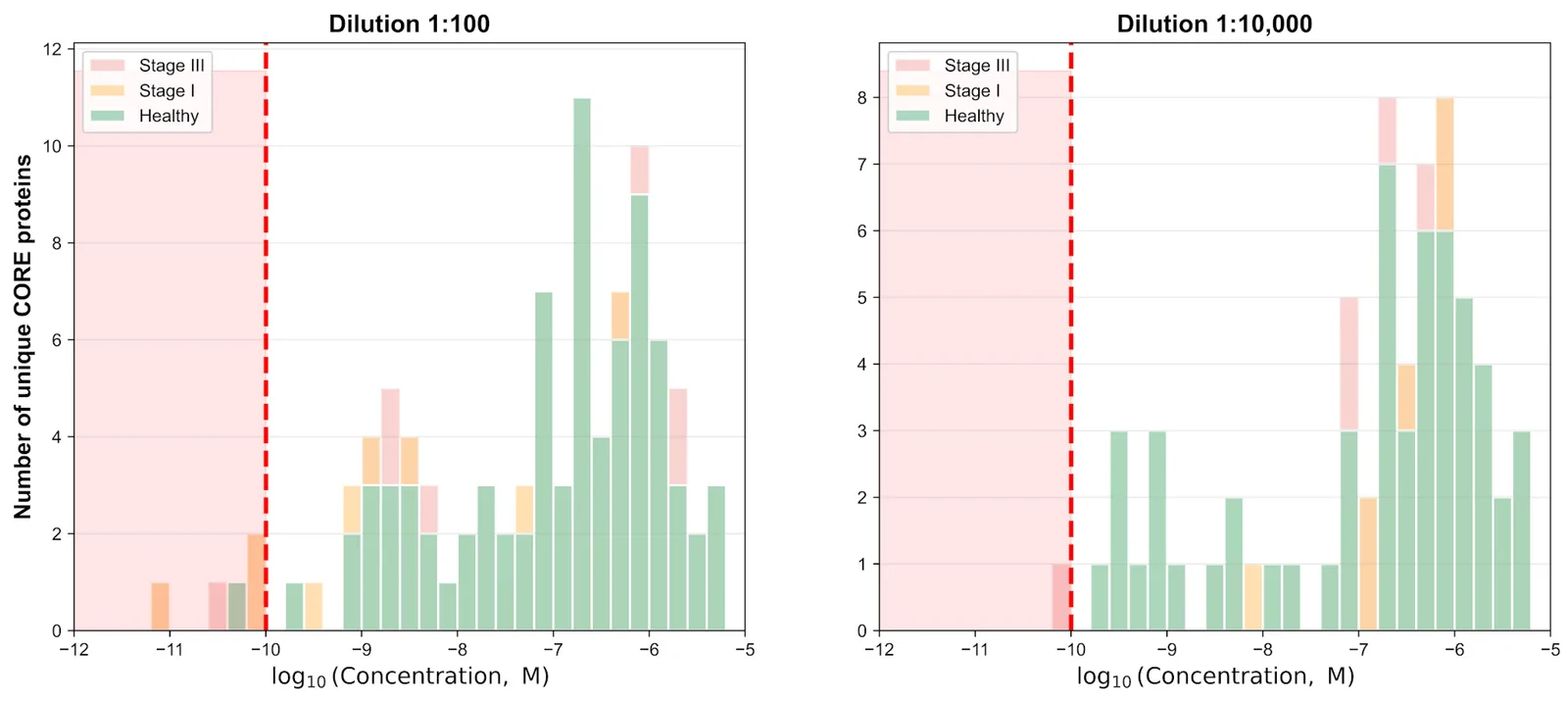
Distribution of reference concentrations of CORE proteins. Histogram showing the distribution of CORE proteins according to log_10_-transformed molar concentrations for the Healthy, Stage I, and Stage III groups at 1:100 and 1:10,000 dilutions. The dashed vertical line indicates the low-abundance threshold at log_10_ concentration = −10. Concentration values represent reference plasma concentrations obtained from the mass-spectrometry-derived dataset in the Blood Protein Atlas section of the Human Protein Atlas and were not measured experimentally in this study. Proteins for which no corresponding concentration annotation was available were excluded from this analysis.

**Figure 5:**
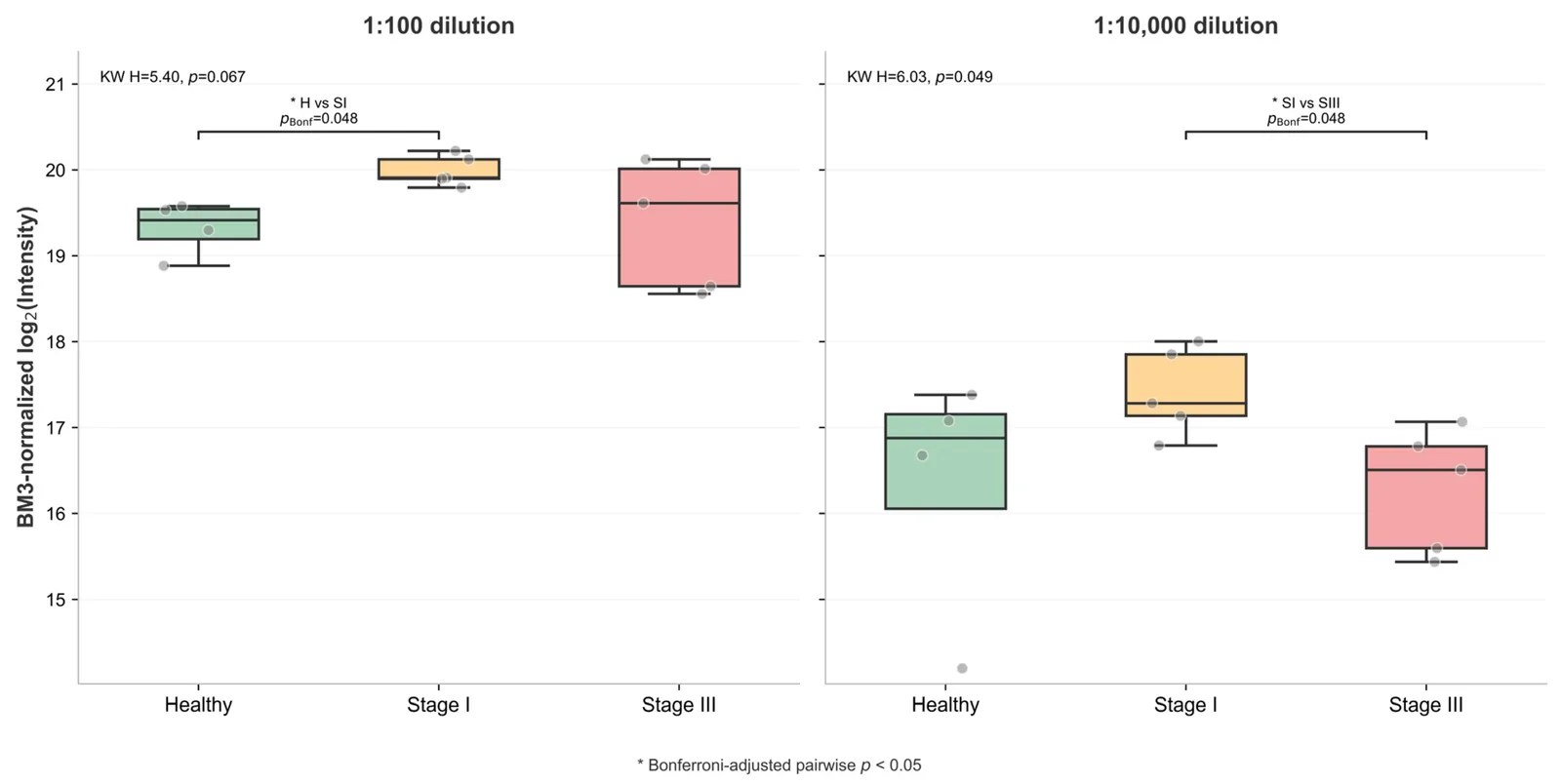
Dilution-dependent behavior of BM3-normalized albumin intensity. Boxplots show BM3-normalized, log_2_-transformed albumin intensities for the Healthy, Stage I, and Stage III groups at 1:100 and 1:10,000 dilutions. Points represent individual samples, and boxes indicate the median and interquartile range. Kruskal–Wallis omnibus results and Bonferroni-adjusted Mann–Whitney pairwise comparisons are shown. Significant pairwise differences were observed between Healthy and Stage I at 1:100 dilution (*p*Bonferroni = 0.048) and between Stage I and Stage III at 1:10,000 dilution (*p*Bonferroni = 0.048). An asterisk (*) indicates a Bonferroni-adjusted pairwise comparison with *p* < 0.05. Significant pairwise differences were observed between Healthy and Stage I at 1:100 dilution (pBonferroni = 0.048) and between Stage I and Stage III at 1:10,000 dilution (pBonferroni = 0.048).

Protein concentration distributions were analyzed for CORE proteins using log_10_-transformed molar concentration values (Figure 4). The values shown in Figure 4 represent reference plasma concentrations obtained from the mass-spectrometry-derived plasma concentration dataset in the Blood Protein Atlas section of the Human Protein Atlas and were not measured directly in the samples analyzed in this study. Accordingly, each protein was assigned the same reference concentration across groups and dilution conditions whenever an Atlas annotation was available. Proteins lacking corresponding concentration data in the Human Protein Atlas, including albumin, were excluded from this analysis. Experimental changes in albumin intensity are presented separately in Section 2.2.4 and Figure 5.

At 1:100 dilution, CORE proteins spanned a broad dynamic range, with log_10_ concentrations approximately from −11.16 to −5.24. The majority of proteins were located in the intermediate range (−8 to −6), while a smaller subset was observed at lower concentrations (≤−10). At 1:10,000 dilution, the total number of CORE proteins decreased, and the distribution was more concentrated within higher log_10_ concentration ranges.

The number of proteins in the low-concentration region (log_10_ ≤ −10) was reduced compared to the 1:100 condition. Low-abundance CORE proteins (log_10_ ≤ −10) were identified in both dilution regimes. At 1:100 dilution, these included MYH4 (−10.20) in Healthy samples; ATP1A1 (−11.16), MYH4 (−10.20), HTRA1 (−10.09) and DSC3 (−10.05) in Stage I; and ATP1A1 (−11.16), PKP1 (−10.49), HTRA1 (−10.09) and DSC3 (−10.05) in Stage III.

At 1:10,000 dilution, only DMBT1 (−10.19) in Stage III samples met the low-abundance criterion. TGM3 (−9.71) and DSC1 (−9.54) in Healthy samples were classified as medium abundance (−10 to −8).

Proteins were classified based on log_10_-transformed concentrations into low (≤−10), medium (−10 to −8), and high (>−8) abundance categories. Across all groups, the majority of CORE proteins were located in the medium and high abundance ranges, whereas the number of low-abundance proteins was limited and decreased at higher dilution.

These results describe a dilution-dependent reduction in the number of CORE proteins observed in the lower range of log_10_ concentrations and a relative concentration of reproducibly detected proteins within higher concentration intervals.

At 1:100 dilution, CORE proteins span a broader range of log_10_ concentrations, including values ≤−10. At 1:10,000 dilution, fewer CORE proteins are observed in this range, while the majority are distributed at higher concentration values.

A subset of low-abundance CORE proteins identified in this study, including ATP1A1, DMBT1, PKP1, MYH4, HTRA1 and DSC3, has been described in the literature in the context of membrane-associated processes, intercellular junctions and extracellular signaling [26,27,28,29,30].

Their presence in plasma samples, particularly in disease groups, may be associated with the release of intracellular or membrane-associated components into circulation under pathological conditions. For example, ATP1A1 has been linked to membrane integrity and signaling pathways, while DMBT1 has been reported in studies related to immune responses and cancer-associated processes [31]. Similarly, proteins such as PKP1 and DSC3 are components of cell–cell junctions and may appear in plasma in association with changes in tissue structure.

At the same time, the detection of these proteins at low concentrations may also reflect analytical factors related to detectability at the lower boundary of the dynamic range of plasma proteomics [32].

Thus, the observed patterns may reflect a combination of biological and analytical effects, and further validation is required to determine the specific contribution of each factor.

#### 2.2.4. Effect of Dilution on Albumin Signal

Albumin (CON__P02768-1; ≈69 kDa, 5.1–7.2 × 10^−4^ mol·L^−1^ in plasma) is the most abundant plasma protein and strongly influences proteomic measurements due to its high concentration and dominance within the dynamic range.

At 1:100 dilution, high BM3-normalized log_2_-transformed albumin intensities were observed across all groups. The median values were 19.41 (IQR 19.19–19.54) in Healthy controls, 19.91 (IQR 19.90–20.12) in Stage I, and 19.61 (IQR 18.64–20.01) in Stage III. Group differences were assessed using the Kruskal–Wallis test, followed by pairwise Mann–Whitney U tests with Bonferroni correction for the three pairwise comparisons. The omnibus test did not reach statistical significance (Kruskal–Wallis statistic, *H* = 5.40; *p* = 0.067). In the pairwise analysis, the Healthy versus Stage I comparison yielded an unadjusted *p*-value (*p*raw) of 0.016 and remained significant after Bonferroni correction (*p*Bonferroni = 0.048). Within-group variability was relatively low in the Healthy and Stage I groups but higher in Stage III, as reflected by the wider IQR.

At 1:10,000 dilution, BM3-normalized albumin intensity decreased in all groups. The median values were 16.88 (IQR 16.06–17.15) in Healthy controls, 17.28 (IQR 17.14–17.85) in Stage I, and 16.51 (IQR 15.60–16.78) in Stage III. Relative to the corresponding medians at 1:100 dilution, albumin intensity decreased by 2.53 log_2_ units in Healthy controls, 2.63 log_2_ units in Stage I, and 3.10 log_2_ units in Stage III, corresponding to 5.78-, 6.19-, and 8.57-fold reductions, respectively. The omnibus Kruskal–Wallis test reached statistical significance (*H* = 6.03; *p* = 0.049). The Stage I versus Stage III pairwise comparison yielded *p*raw = 0.016 and remained significant after Bonferroni correction (*p*Bonferroni = 0.048). Within-group dispersion increased in the Healthy and Stage I groups at this dilution.

In contrast, the Stage III group retained a relatively broad distribution under both dilution conditions. Although the median albumin intensity decreased from 19.61 at 1:100 dilution to 16.51 at 1:10,000 dilution, the IQR remained comparatively wide (18.64–20.01 and 15.60–16.78, respectively). This suggests that the dispersion observed in Stage III samples was not solely attributable to the reduction in signal intensity.

Overall, dilution affects both signal magnitude and variance structure. In more homogeneous groups (Healthy and Stage I), reduced signal is associated with increased relative noise. In Stage III, variability remains consistently high across conditions, suggesting a substantial contribution of within-group heterogeneity.

These results indicate that the albumin signal does not behave as a stable reference across conditions. Its intensity and variability depend on both signal level and group structure. Therefore, normalization to albumin may introduce bias, particularly when comparing groups with different variability patterns, and should be avoided in this experimental setting.

At 1:100 dilution, albumin shows uniformly high signal with limited separation between groups. At 1:10,000 dilution, intensity decreases and group differences become more apparent, but this is accompanied by increased variability in Healthy and Stage I. In contrast, Stage III remains consistently variable across both conditions, reflecting persistent within-group heterogeneity.

#### 2.2.5. Differentially Abundant Proteins

Differential abundance analysis was performed separately for each dilution condition to identify disease-associated changes in the plasma proteome between Stage III ovarian cancer patients and healthy controls. The analysis was restricted to a curated set of reviewed human proteins (UniProt Swiss-Prot entries), ensuring high annotation quality and biological interpretability.

At 1:100 dilution, 11 proteins met the nominal exploratory criterion (|log_2_FC| > 1, unadjusted *p* < 0.05). Of these, only RARRES2 and BASP1 remained significant after Benjamini–Hochberg correction (FDR < 0.05), whereas the remaining proteins were considered hypothesis-generating candidates. Among the nominal candidates, 3 proteins were upregulated in Stage III, including RARRES2 (chemerin), BASP1, and ITIH3. The majority of proteins (8 out of 11) were downregulated, predominantly representing classical high-abundance plasma components. These included complement proteins (C3, C8G), protease inhibitors (SERPINC1, SERPINF1), the antimicrobial enzyme LYZ, as well as IGHA1, FBLN1, and PON1. This pattern indicates a general decrease in several key circulating proteins involved in immune response, protease regulation, and extracellular matrix stability.

In contrast, at 1:10,000 dilution, six proteins met the nominal exploratory criterion (|log_2_FC| > 1, unadjusted *p* < 0.05), but none remained significant after Benjamini–Hochberg correction; these proteins were therefore considered hypothesis-generating candidates. Five of the six proteins showed increased abundance in Stage III samples and were predominantly immunoglobulin-related, including IGKC, IGLV1-47, IGKV3D-11, and IGKV2-24, together with SERPING1 (C1 esterase inhibitor). F5 (coagulation factor V) was the only protein showing decreased abundance at this dilution. No proteins were shared between the nominal candidate sets identified at the two dilution conditions. At 1:100 dilution, the candidate set was predominantly composed of abundant circulating plasma proteins, whereas the 1:10,000 set contained a higher proportion of immunoglobulin-related proteins. These findings are consistent with a dilution-dependent change in the observable fraction of the plasma proteome and suggest that the differences between the two candidate sets reflect both biological variation and the analytical effects of sample dilution.

Given the limited sample size (*n* = 4–5 per group) and the exploratory nature of this pilot cohort, these findings should be interpreted as indicative rather than definitive. Validation in larger independent cohorts will be required to confirm the observed patterns and assess their potential clinical relevance.

**Figure 6:**
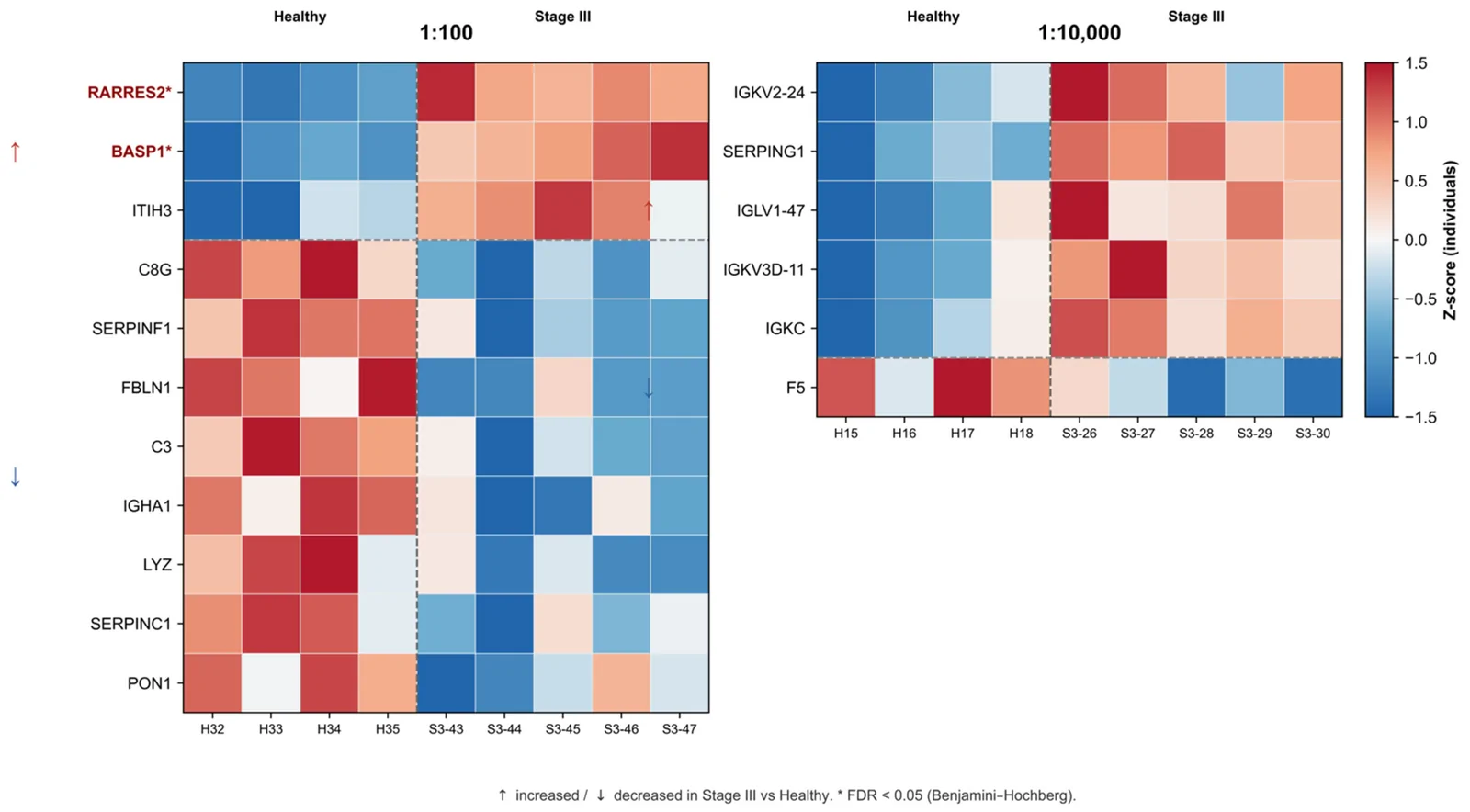
Heatmap of differential protein abundance between Healthy controls and Stage III ovarian cancer patients, shown for individual samples. H, Healthy controls; S3, Stage III ovarian cancer. Red upward arrows (↑) mark proteins increased in Stage III relative to Healthy controls; blue downward arrows (↓) mark proteins decreased in Stage III. The thick vertical line separates dilution conditions; thick horizontal lines separate protein groups detected at different dilutions; dashed lines indicate separation between up- and downregulated proteins within each dilution group. Gray cells indicate proteins that were not quantified in the corresponding samples, rather than proteins that merely failed to meet the significance threshold. FDR-significant proteins (RARRES2 and BASP1, at 1:100 dilution only) are highlighted. Complete differential-expression statistics are reported in Table S1 (Supplementary Materials).

Figure 6 shows heatmap of Z-scored individual-sample protein intensities for nominal exploratory candidates identified at two plasma dilution conditions: 1:100 (left panel, *n* = 11 proteins) and 1:10,000 (right panel, *n* = 6 proteins).

Candidate proteins were defined by |log_2_FC| > 1 and an unadjusted *p* < 0.05. Individual samples are shown separately to illustrate within-group variability. Proteins are arranged according to dilution condition and the direction of change in Stage III ovarian cancer relative to Healthy controls. Heatmap values represent per-protein Z-scores calculated from log_2_-transformed intensity values, with blue indicating lower relative abundance and red indicating higher relative abundance. Gray cells denote proteins that were not quantified in the corresponding samples or were not retained in the respective dilution-specific analysis matrix. RARRES2 and BASP1 at 1:100 dilution were the only proteins that remained significant after Benjamini–Hochberg correction (FDR < 0.05) and are highlighted accordingly; no proteins at 1:10,000 dilution reached FDR significance. No proteins were shared between the nominal candidate sets identified at the two dilution conditions. At 1:100 dilution, 3 proteins showed increased and 8 showed decreased abundance in Stage III samples, whereas at 1:10,000 dilution, 5 proteins showed increased and 1 showed decreased abundance. Complete protein annotations, fold changes, nominal *p*-values, and FDR-adjusted *p*-values are provided in Zenodo repository.

## 3. Discussion

The AFM imaging data are consistent with our previously obtained data for healthy volunteers, where AFM imaging confirmed the effective concentration of proteins on the surface modified with a photocrosslinker [25].

MS results indicate that sample dilution has a strong effect on the detected differential plasma proteome and on the interpretation of differences between healthy controls and patients. In the case of 1:100 plasma dilution, most altered proteins show decreased abundance in Stage III. These proteins represent major plasma components, including complement proteins, protease inhibitors, and antimicrobial proteins. This reflects changes in circulating protein composition related to immune and proteolytic regulation. In the case of 1:10,000 plasma dilution, an opposite pattern is observed, with an increased contribution of immunoglobulin-related proteins. Both constant and variable immunoglobulin regions become detectable, along with a complement regulator, while coagulation factor V shows decreased abundance.

The mechanism determining the varying composition of proteins immobilized on the chip surface at different sample dilutions appears quite complex and is not yet fully understood. At present, we can only hypothesize that it results from a combination of several interconnected processes. We attribute this to the selectivity of benzophenone for certain amino acids (Met, Leu, Gly, etc.), steric limitations of the reaction (distance of ~3 Å), and hydrophobic interactions that ensure the preliminary concentration of proteins on the surface. It is possible that the Vroman effect—the replacement of primarily adsorbed albumin by proteins with higher affinity—occurs before photofixation. Furthermore, sample dilution likely promotes the dissociation of protein aggregates and oligomers, increasing the proportion of monomeric forms, which are more accessible for adsorption and photoimmobilization.

Overall, the composition of the detectable protein subset differed between dilution conditions. Proteins related to the complement system, protease regulation, coagulation, and immunoglobulin fractions appear to be the most informative. The combined use of both dilution conditions provides a more complete description of plasma proteome changes.

When interpreting these results, it is important to consider analytical factors influencing protein detection at different plasma dilutions. The distribution of proteins by molecular mass showed dominance of the 30–100 kDa range at 1:100 dilution (52–53% of total), corresponding to the most abundant circulating plasma proteins. At 1:10,000 dilution, the total number of CORE proteins decreased and the relative contribution of low-molecular-weight proteins was reduced, indicating a dilution-dependent shift in the composition of the reproducibly detected protein subset. Analysis of albumin stability as a reference protein revealed its limited suitability for data normalization: upon transition to 1:10,000 dilution, the median albumin signal decreased by 2.53, 2.63, and 3.10 log_2_ units in the Healthy, Stage I, and Stage III groups, corresponding to 5.78-, 6.19-, and 8.57-fold reductions, respectively. Dispersion increased in the Healthy and Stage I groups, whereas the Stage III group retained a comparatively broad distribution under both dilution conditions. This may reflect both analytical instability in measuring low-concentration albumin and biological heterogeneity among patients.

Protein expression patterns and tissue specificity were inspected using the Human Protein Atlas [33].

At 1:100 dilution, decreased levels of complement system proteins (C3, C8G) were detected, which may represent consumption of these factors in the tumor microenvironment [34]. The complement system participates not only in anti-infectious defense but also in inflammatory and tumor processes, with complement activation exerting dual effects on tumors—both immunoprotective and growth-promoting through autocrine mechanisms [35]. Reduction in SERPINC1 (antithrombin III) is consistent with data on the prothrombotic state in ovarian cancer: antithrombin deficiency creates conditions for venous thromboembolic complications, which are a leading cause of morbidity in these patients [36,37]. Decreased PON1 indicates impaired antioxidant defense—systematic reviews demonstrate a consistent association between reduced PON1 activity and cancer presence, including ovarian cancer, reflecting oxidative stress in the tumor environment [38]. The increase in RARRES2 (chemerin) is of interest given the complex role of chemerin in carcinogenesis—both decreased expression in tumor tissue and increased levels in circulation have been described, which may reflect compensatory mechanisms or different biological activity of tissue versus circulating forms [39].

At 1:10,000 dilution, the differential protein profile fundamentally differed from the 1:100 dilution case. Elevation of immunoglobulin components (IGKC, IGLV1-47, IGKV3D-11, IGKV2-24) may reflect polyclonal B-cell activation or presence of tumor-associated immunoglobulin components. Increased SERPING1 (C1-inhibitor) in the context of decreased C3 and C8G at 1:100 dilution may represent compensatory regulation of the complement system [35]. Decreased F5 (coagulation factor V) is consistent with the concept of consumption of coagulation factors during microthrombus formation in malignancies [36]. The two dilution conditions yielded partially different protein subsets, indicating dilution-dependent selectivity and complementary proteome coverage. Importantly, 16 CORE proteins detected at 1:10,000 were absent from the 1:100 CORE union, increasing the combined CORE set from 216 to 232 proteins. Despite differences, both profiles converge on four interconnected systems: complement, coagulation, proteases, and immune response.

Thus, the approach we developed—combining protein concentration on AFM chip surfaces with subsequent mass spectrometric identification—has demonstrated its effectiveness in detecting a wide range of plasma proteins, including low-abundance components that remain undetectable via standard analysis. We anticipate that this method will prove valuable when applied to larger patient cohorts, enabling the identification of potential biomarkers for the early stages of major diseases. It is also worth noting that the AFM chip platform is not limited to the proposed non-specific concentration scheme; a biospecific recognition approach can be implemented by immobilizing antibodies or aptamers on the surface, thereby allowing for the targeted isolation of specific protein targets. Furthermore, such an antibody-based setup could be used to partially deplete major components from complex biological samples. While we do not view this as a replacement for classical immunodepletion methods, it could serve as a useful auxiliary tool that complements existing enrichment strategies. Looking ahead, we plan to further develop the method—including expanding the range of AFM-based characteristics and integrating it with other approaches—which we expect will enhance the sensitivity and informational value of proteomic profiling.

## 4. Materials and Methods

### 4.1. Study Design and Samples

The study included plasma samples from 14 participants divided into three groups: healthy donors (*n* = 4) [25], patients with stage I ovarian cancer (*n* = 5), and patients with stage III ovarian cancer (*n* = 5). Plasma samples were obtained from women aged 51 to 54 years. Each sample was analyzed at two dilution levels—1:100 and 1:10,000—in multiple technical replicates (see Section 4.7.1 for replicate numbers per sample type), which allowed us to evaluate the effect of sample concentration on the depth of proteome coverage.

The study was approved by the independent ethics committees of the organizations that provided the samples (Protocol No. 258 of 10 August 2021). All patients provided written informed consent to participate in the study and for the use of their biological material.

### 4.2. Experimental Design

**Figure 7:**
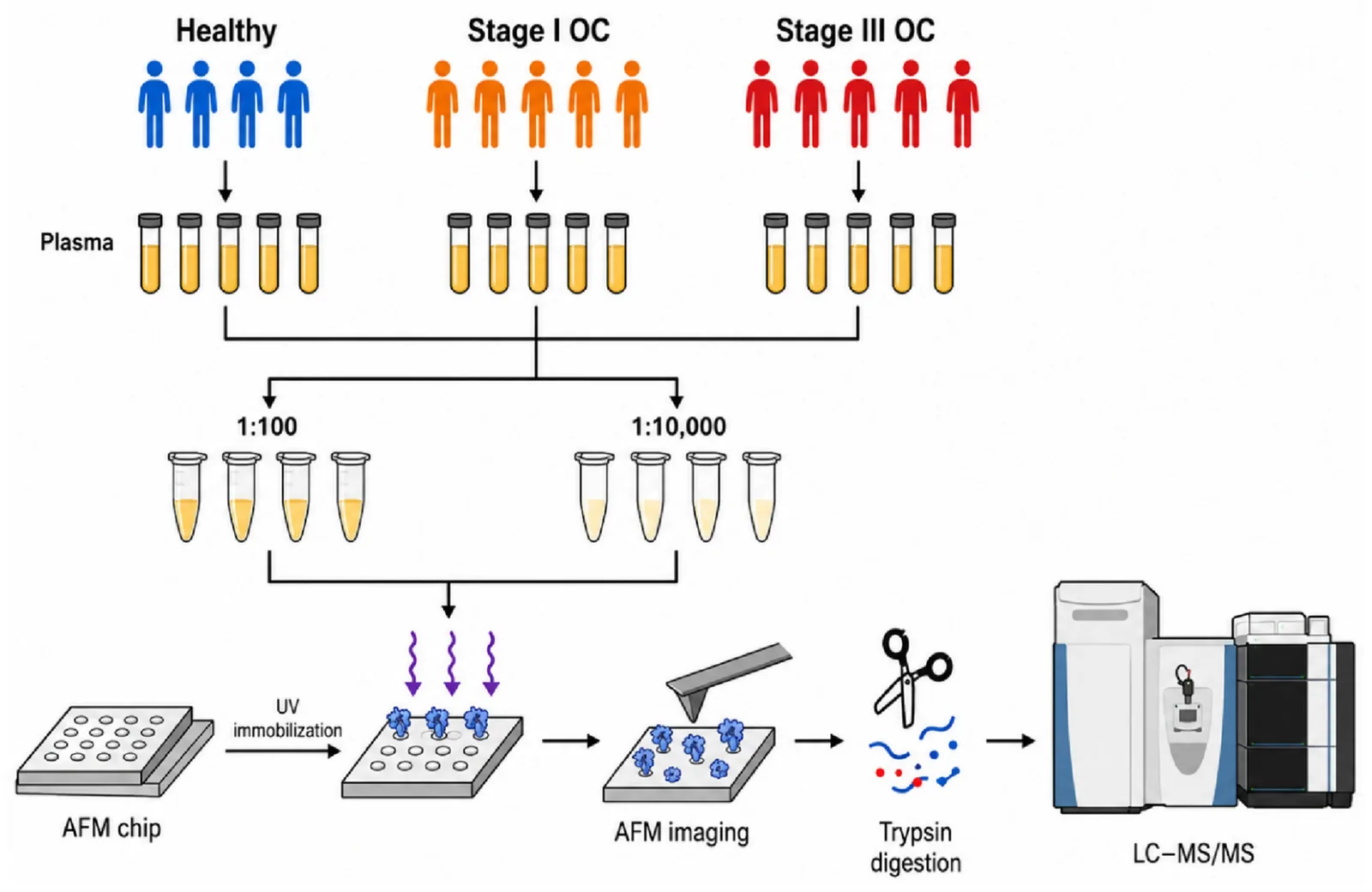
Experimental design scheme. Plasma samples from Healthy (control), Stage I, and Stage III ovarian cancer groups were diluted 1:100 or 1:10,000 and incubated on SuccBB-modified AFM chips under UV irradiation for protein immobilization. Chips were imaged by AFM and then digested with trypsin, and the resulting peptides were analyzed by LC-MS/MS.

The experimental design is shown in Figure 7.

Step 1. Plasma samples were collected from healthy donors and from patients with stage I and stage III ovarian cancer.

Step 2. Plasma samples were diluted 1:100 and 1:10,000. Samples from healthy donors served as the control group, while samples from patients with stage I and stage III ovarian cancer constituted the disease groups; all samples were processed identically in subsequent steps.

Step 3. AFM chips with SuccBB-modified surfaces were incubated in diluted plasma (1:100 or 1:10,000) under UV irradiation to immobilize plasma proteins on the chip surface.

Step 4. All chips were scanned by AFM after incubation.

Steps 5 and 6. AFM chips with concentrated plasma proteins were digested with trypsin. Eluates were analyzed by LC-MS/MS.

### 4.3. Modification and Activation of the AFM Substrate Surface

Muscovite mica substrates (SPI, West Chester, PA, USA) were cut into 7.5 × 15 mm pieces. The substrate surface was modified by (3-aminopropyl)triethoxysilane (APTES) (Acros Organics, Fair Lawn, NJ, USA) by vapor-phase deposition [40].

SuccBB (Sigma, St. Louis, MO, USA) solution (0.31 mM) was prepared in DMSO (Servicebio, China). The AFM substrate was incubated in the activating solution with shaking (120 rpm) for 18 h at room temperature. The substrate was washed once with 1 mL DMSO:EtOH (1:1) at 40 °C for 30 min and then twice with 1 mL 50% EtOH for 30 min each. The chip was dried under nitrogen.

### 4.4. Incubation for Plasma Protein Immobilization

The SuccBB-activated AFM substrate was incubated with 1 mL of plasma at the corresponding dilution (1:100 or 1:10,000) in a rotating tube at 200 rpm under UV irradiation for 1 h. UV irradiation was performed using a UVP Crosslinker CL-3000L device (Analytik Jena US, Upland, CA, USA) at 365 nm wavelength. The distance between the tube and UV source was approximately 10 cm.

After incubation, chips were washed once with 1 mL of 0.01% aqueous Emulgen 913 (Kao Atlas, Osaka, Japan) at 37 °C for 30 min and then twice with 1 mL ultrapure water at 37 °C for 30 min each, and then air-dried.

### 4.5. AFM Scanning

AFM chip surfaces were scanned in semi-contact mode using Titanium or Solver Next atomic force microscopes (NT-MDT, Zelenograd, Russia). NSG10 cantilevers (TipsNano, Zelenograd, Russia) were used with tip curvature radius ~10 nm, resonance frequency 140–390 kHz, and force constant 3.1–37.6 N/m. Scan size was 5 × 5 µm^2^ (resolution 256 × 256 points). At least 10 scans of different surface areas were obtained for each substrate.

AFM images were processed using NovaPx 3.5.0 software, including second-order plane subtraction. Object size was determined by height.

### 4.6. Sample Preparation for Mass Spectrometry

For on-chip trypsinolysis, AFM chips with immobilized plasma proteins were immersed in a solution containing 10 µL of trypsin (Promega Corp., Cat. # V5111, Madison, WI, USA) solution (0.18 g/L), 20 µL of TEAB (Honeywell Fluka, Washington, DC, USA) buffer (50 mM), and 600 µL of deionized water to completely cover the mica surface. Trypsinolysis of surface-immobilized proteins was performed on an incubator shaker at 37 °C and 140 rpm for 18 h, followed by incubation in a heat chamber at 40 °C for 2 h in a horizontal position. The chips in the tryptic mixture were then shaken at 40 °C for 60 min, vortexed using a SkyLine vortex mixer (Elmi Ltd., Rīga, Latvia) in MIX1 mode, and centrifuged at 9000 rpm for 10 min. Hydrolysis was stopped by adding 100 µL of 0.1% formic acid. No reduction or alkylation was performed for the AFM-chip samples; proteins immobilized on the SuccBB-functionalized surface were subjected directly to enzymatic digestion with trypsin.

The BM3 calibration protein was prepared separately and was not added during on-chip trypsinolysis. BM3 was subjected to independent trypsin digestion by incubating 300 µL of BM3 solution in 50 mM TEAB with 15 µL of trypsin solution (0.18 g/L). After digestion, 10 µL of the trypsin-treated BM3 solution at a concentration of 10^−7^ M was added to each peptide mixture, corresponding to 1 pmol of digested BM3 per sample. BM3-derived peptides were used as an internal standard for quality control and semi-quantitative normalization.

After addition of the BM3 digest, peptide mixtures were dried in a vacuum centrifuge SpeedVac (Eppendorf, Macquarie Park, NSW, Australia) and then dissolved in 5% (*v*/*v*) formic acid for LC-MS/MS analysis.

### 4.7. MS Measurements

#### 4.7.1. LC-MS Measurements

Proteomic analysis was performed using a Q Exactive HF Hybrid Quadrupole-Orbitrap mass spectrometer (Thermo Fisher Scientific, Rockwell, IL, USA). Peptides were eluted with a 90-min gradient, as previously described. For each eluate from AFM chips, at least three technical replicates were measured, including samples from the Healthy (control), Stage I, and Stage III groups.

#### 4.7.2. Acquisition of MS Data

Mass spectrometric measurements were performed as previously described [41] using a Q Exactive HF mass spectrometer (Thermo Fisher Scientific™, Rockwell, IL, USA). Mass spectra were obtained with resolution of 60,000 (MS) and 15,000 (MS/MS) in the *m*/*z* range 400–1500 (MS) and 200–2000 (MS/MS).

#### 4.7.3. MS Data Processing

Data were processed using MaxQuant (version 2.2.0.0; Jürgen Cox, Max Planck Institute of Biochemistry, Martinsried, Germany) [42,43]. Carbamidomethylation of cysteine, N-terminal protein acetylation, and methionine oxidation were set as variable modifications. The false discovery rate (FDR) was set at 0.01. A maximum mass deviation of 5 ppm was allowed for precursor identification and 20 ppm for fragment identification. For trypsin digestion, up to two missed cleavage sites were allowed. Protein identification was performed against the reviewed human UniProt proteome database [44], supplemented with BM3 protein.

**Normalization to BM3 calibration protein.** Protein-level iBAQ intensity values were obtained from the MaxQuant proteinGroups output. For each technical LC–MS/MS measurement, protein iBAQ values were first normalized to the corresponding BM3 iBAQ value. For each participant and protein, the maximum of the three BM3-normalized technical-replicate values was then retained as a single participant-level value. All subsequent statistical analyses were performed using these participant-level values; technical replicates were not treated as independent observations.

**Removal of contaminants.** Contaminant proteins such as keratin were excluded from processing. Signals recorded in control samples (blank AFM chips processed without plasma) were subtracted from all experimental samples to remove background noise and cross-contamination.

**CORE proteome selection.** For each group (Healthy, Stage I, and Stage III) and dilution condition (1:100 and 1:10,000), the CORE proteome was defined as the intersection of proteins with participant-level intensity greater than zero in every participant column of that group. Accordingly, the Healthy CORE was defined across four participants, whereas the Stage I and Stage III CORE sets were each defined across five participants.

Proteins were considered detected if at least two unique peptides were present.

### 4.8. Statistical Analysis

#### 4.8.1. Descriptive Statistics and Reproducibility

For each group, mean number of detected proteins, standard deviation, and coefficient of variation (CV, %) were calculated. Overlap of identified proteins was assessed using Union and CORE metrics.

#### 4.8.2. Participant-Level Data and Statistical Units

All inferential statistical analyses were performed at the level of independent participants (Healthy, *n* = 4; Stage I, *n* = 5; Stage III, *n* = 5). Missing, non-finite, or non-positive intensity values were treated as not quantified; no imputation was performed.

#### 4.8.3. Albumin Group Comparisons

For albumin intensity (Section 2.2.4, Figure 5), descriptive statistics are reported as the median and interquartile range (IQR), consistent with the nonparametric approach used for group comparisons. At each dilution, an omnibus Kruskal–Wallis test was performed across the three groups (Healthy, Stage I, and Stage III), followed by pairwise Mann–Whitney U tests for the three between-group comparisons. Both nominal and Bonferroni-adjusted *p*-values are reported for the pairwise comparisons, whereas the Kruskal–Wallis *p*-value is reported separately as the omnibus test result. Albumin intensity was not used for normalization.

### 4.9. Differential Protein Analysis

To identify proteins differing in abundance between the Healthy and Stage III groups, Welch’s two-sample t-test was performed separately at each dilution (1:100 and 1:10,000) using participant-level BM3-normalized iBAQ values (Healthy, *n* = 4; Stage III, *n* = 5). Proteins were tested only when at least two positive quantified values were available in each group; missing, non-finite, and non-positive values were not imputed. Two levels of statistical evidence were considered: (i) nominal exploratory candidates, defined by |log_2_FC| > 1 and an unadjusted *p* < 0.05; and (ii) FDR-significant proteins, defined as proteins additionally meeting the Benjamini–Hochberg false discovery rate criterion (FDR < 0.05). Nominal candidate lists are treated as hypothesis-generating, whereas only proteins satisfying the FDR criterion are considered statistically significant.

### 4.10. Software

Statistical analysis was performed in Python 3.12 using pandas (v2.0), numpy (v1.24), scipy (v1.11), and statsmodels (v0.14). Data visualization was performed using matplotlib (v3.8) and seaborn.

## 5. Conclusions

In this study, the AFM/MS method was applied to analyze plasma samples from patients with early- and late-stage ovarian cancer using a small cohort (4–5 samples per group). The use of two dilution levels—1:100 and 1:10,000—provided complementary information on protein composition. The lower dilution revealed a greater number of proteins belonging to the “proteome core”—defined as proteins reproducibly identified across all samples in a group—whereas the higher dilution altered the profile of detectable proteins. Dilution also affected albumin behavior, with decreasing signal intensity and increasing variability indicating that albumin is unsuitable as a stable reference for normalization under these conditions. The identified proteins represented various classes, including immunoglobulins, acute-phase proteins, regulatory proteins, and complement system components. The method is notable for its streamlined experimental workflow. A flat chip enables all analytical stages—surface modification, sample incubation, AFM imaging, and enzymatic hydrolysis—to be performed sequentially on a single substrate, thereby minimizing sample loss and measurement variability. The platform also offers adaptability; for instance, antibodies or aptamers can be immobilized to enable biospecific isolation of target proteins or partial depletion of major components. Despite limitations related to the small sample size and the lack of orthogonal validation, these results provide a foundation for future studies in larger cohorts aimed at identifying biomarkers for diseases of substantial clinical relevance.

Sample dilution represents an important experimental parameter in AFM/MS analysis, as it influences the composition of the detectable plasma proteome. The use of different dilution conditions may therefore provide complementary information on different observable fractions of the plasma proteome. Further studies in larger independent cohorts, together with orthogonal analytical validation, are required to assess the robustness and potential utility of this approach for biomarker discovery.

## Data Availability

The mass spectrometry proteomics data generated in this study have been deposited to the ProteomeXchange Consortium via the PRIDE partner repository under the dataset identifier PXD068982. The analysis code and key result tables supporting the findings of this study are publicly available via Zenodo at https://zenodo.org/records/22050808, accessed on 19 August 2026.

## Supplementary Materials

The following supporting information can be downloaded at: https://www.mdpi.com/article/10.3390/ijms27177541/s1.
