# Supplementary material for: Blood Plasma Analysis in Ovarian Cancer Patients Using an AFM/MS Approach: Effect of Sample Dilution on Proteome Depth
Source: Int J Mol Sci. 2026 Aug 23;27(17):7541. doi: 10.3390/ijms27177541 (PMC13565955; doi:10.3390/ijms27177541)
Supplement: Supplementary file 1 [file ijms-27-07541-s001.zip › Supplementary_IJMS/Supplementary_Materials.pdf]

Supplementary Materials

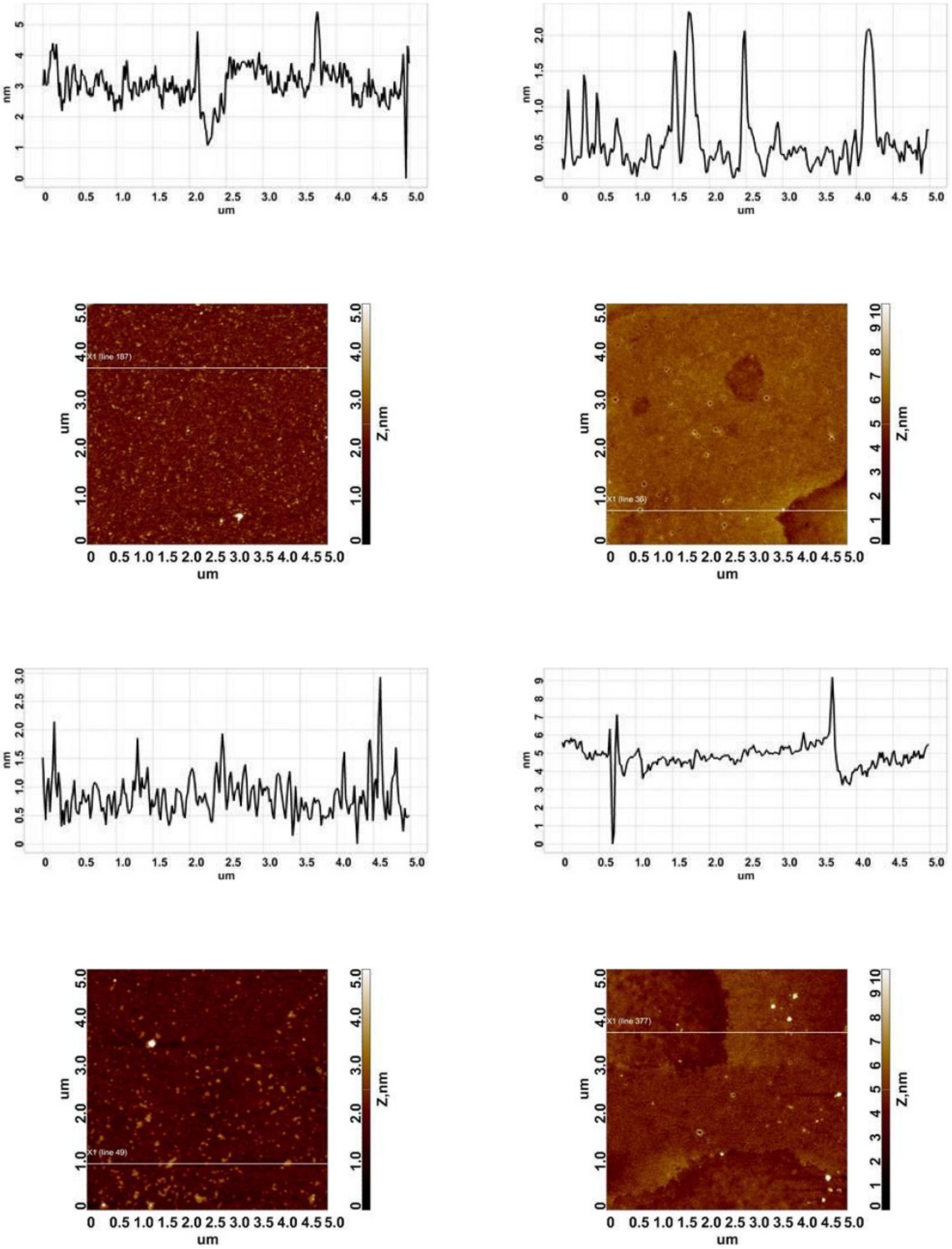

Figure S1. Typical AFM images of the surface of modified mica after incubation in plasma of patients with stages I and III: (A,B) stage I, dilution 1:100 (scan and section); (C,D) stage I,

dilution 1:10,000 (scan and section); (E,F) stage III, dilution 1:100 (scan and section); (G,H) stage III, dilution 1:10,000 (scan and section). The size of AFM images is 5 × 5 μm^2.

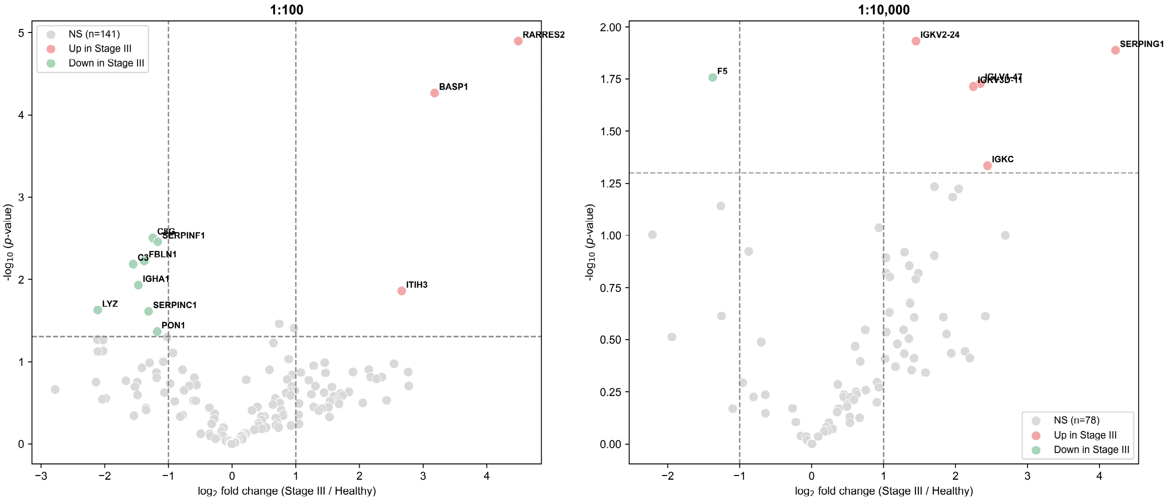

Figure S2. Volcano plots of differential protein abundance between Stage III ovarian cancer patients and Healthy controls at 1:100 (left) and 1:10,000 (right) dilution. Proteins with |log2FC| > 1 and $p < 0.05$ are labelled. Complete statistics are in Table S1.

Table S1. Complete differential-expression statistics for all nominal candidate proteins at 1:100 and 1:10,000 dilution (log2 fold change, nominal p-value, and Benjamini–Hochberg FDR).

File: Table_S1.xlsx

Table S2. Molecular mass and reference plasma abundance annotations for all quantified proteins.

File: Table_S2.xlsx
